# Machine Learning Is Not Just for Prediction: Its Role as an Exploratory Analytical Tool in Medicine

**DOI:** 10.3400/avd.ra.26-00055

**Published:** 2026-06-17

**Authors:** Sei Komatsu

**Affiliations:** Department of Cardiology, Cardiovascular Center, Osaka Gyoumeikan Hospital, Osaka, Osaka, Japan

**Keywords:** exploratory analytical tools, hypothesis generation, machine learning, medical statistics

## Abstract

Machine learning (ML) has been primarily used for predictive modeling in medical research, but this reflects only part of its potential. This review proposes a conceptual distinction between predictive ML and exploratory ML. Predictive ML aims to maximize accuracy on unseen data. Exploratory ML focuses on identifying underlying structures in data to generate hypotheses. Exploratory ML plays an important role under conditions where conventional hypothesis-driven statistics have limitations, including high-dimensional data, small sample sizes, and biopsy-inaccessible organs such as the vascular system. Because ML-derived results are based on associations rather than causality, they should be interpreted as hypothesis-generating rather than confirmatory. Methods including unsupervised learning, interpretable supervised learning, and network analysis are discussed as exploratory ML approaches. The differences in objectives and evaluation criteria between exploratory ML and conventional hypothesis-driven statistics are also discussed, together with the structural gap in peer review. The key argument is that studies using exploratory ML should be evaluated not by predictive performance but by the stability, reproducibility, and interpretability of the identified structures. Without this shift, exploratory analyses may be systematically misjudged within current evaluation standards. This perspective may bridge exploratory analysis and confirmatory research and support new study designs in medicine.

## Introduction

High-dimensional datasets have become increasingly common in medical research. The number of parameters often far exceeds what conventional statistical frameworks were originally designed to handle in fields such as genomics and large-scale clinical data analysis.^1,2)^ Traditional hypothesis-driven statistical methods may face fundamental limitations under conditions with small sample sizes, imbalanced data, and high-dimensional molecular data.^3,4)^ Machine learning (ML) has emerged as an alternative analytical method in such settings. However, this dominant view is incomplete and has shaped how ML studies are evaluated in medicine: ML in medicine is predominantly regarded as a tool for predictive modeling.^5–7)^ External validation is often expected, even in studies where ML is used primarily for exploratory purposes. Interpretation and hypothesis generation are also important steps before validation.^8)^ ML-based medical studies may be criticized from both sides. Clinicians may question their interpretability or biological rationale. Methodologists may question whether benchmark gains translate into meaningful clinical progress.^9–11)^ Nevertheless, ML methods are equally capable of functioning as exploratory analytical tools.^8,12)^ In particular, they can be applied to pattern recognition, discovery of variable interactions, and hypothesis generation in domains where a priori hypotheses are absent or premature. This perspective becomes especially important in complex or poorly defined clinical conditions, where conventional hypothesis-driven approaches are difficult to apply. Although clinical researchers often encounter a conceptual gap between traditional statistical methods and ML, this gap itself has rarely been systematically examined. In this narrative review, I redefine ML not only as a tool for predictive modeling but also as a hypothesis-generating analytical framework. Recognizing this distinction is essential for appropriate study design, reporting, and peer review in medical research. This perspective has systematically limited the use of ML’s exploratory potential. This review also aims to integrate existing concepts, including unsupervised learning,^13)^ exploratory data analysis,^14)^ biomarker discovery,^1,8)^ and hypothesis-driven artificial intelligence,^15)^ and to reposition the exploratory role of ML in medicine from a methodological standpoint. ML can be seen as extending exploratory data analysis by enabling the detection of complex structural relationships in high-dimensional biological systems. This distinction also implies that evaluation criteria should be aligned with the primary objective of the analysis.

## Exploratory Data Analysis

The core question is whether hypotheses should precede data analysis or instead emerge from the data. In current medical research, hypothesis-driven study designs remain the dominant approach. A common criticism of ML in medical statistics is that it relies on data-first approaches without hypotheses.^8,16)^ In contrast, Tukey’s concept of exploratory data analysis emphasizes that both exploratory and confirmatory phases are essential in data analysis.^17)^ Importantly, new phenomena are often identified from single cases even in contemporary clinical medicine. Clinicians frequently encounter situations in which previously unrecognized conditions must be organized and interpreted. For example, new disease concepts, such as Kawasaki disease^18)^ or takotsubo cardiomyopathy,^19)^ have been discovered from limited case observations. This process can be understood as an exploratory process in which data precede hypotheses. As argued by Leo Breiman, traditional statistical modeling begins by specifying a data-generating model and its associated distributional assumptions. However, such assumptions may not adequately reflect the complexity of real-world data.^20)^ In the 2000s, large-scale data mining became widespread.^21)^ At the same time, concerns about overreliance on p-values were raised.^22–24)^ As a result, hypothesis generation from data gained wider acceptance. The culture of data visualization in data science can be seen as an extension of this trend.^25)^ Methods such as feature exploration and dimensionality reduction in ML can also be regarded as established approaches related to exploratory data analysis.^26,27)^ Thus, ML extends Tukey’s concept of exploratory data analysis by enabling the detection of complex patterns in high-dimensional data. Exploratory data analysis represents a foundational approach, whereas exploratory ML extends this paradigm by enabling the identification of complex structural relationships. Hypothesis generation in this study should be clearly distinguished from confirmatory inference. This approach should not be mistaken for multiple testing or so-called p-hacking.^28)^ Instead, it should be understood as a process of identifying structural patterns and generating hypotheses. The findings should then be validated using independent datasets.

The ambiguous positioning of exploratory ML has led to its systematic underestimation, particularly within current clinical and data science review frameworks.^29,30)^ In the era of high-dimensional and imbalanced clinical data, new approaches to hypothesis generation are essential.

## Two Types of Machine Learning: Predictive ML and Exploratory ML

**Fig. 1:**
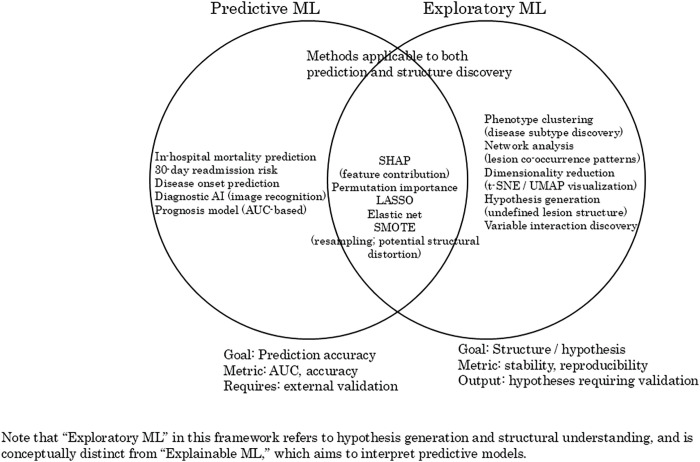
Conceptual framework of predictive and exploratory machine learning. This figure illustrates the conceptual difference between predictive and exploratory uses of ML. The same methods may serve both purposes depending on the analytical objective. ML: machine learning; AI: artificial intelligence; AUC: area under the curve; SHAP: Shapley additive explanations; LASSO: least absolute shrinkage and selection operator; SMOTE: synthetic minority over-sampling technique; t-SNE: t-distributed stochastic neighbor embedding; UMAP: uniform manifold approximation and projection

ML has 2 distinct roles: one is aimed at diagnosis and prediction, and the other is aimed at understanding relationships and structure within data. In this article, I propose a conceptual distinction between predictive ML and exploratory ML (**Fig. 1**). The definitions of these 2 concepts are summarized in **Table 1**. This distinction forms the foundation of this review. In many medical ML studies, exploratory analyses are still discussed mainly in terms of predictive performance, even when the primary aim is structure discovery or hypothesis generation. In this review, I discuss exploratory ML from the viewpoints of structure discovery, hypothesis generation, stability, and interpretability, and I propose that these objectives may require evaluation frameworks different from those used in predictive modeling. This dichotomy does not reject existing classifications; It is introduced as a conceptual framework to clarify the objectives and evaluation criteria of ML research in medicine. Exploratory ML and predictive ML answer different scientific questions. Therefore, applying the same evaluation criteria can lead to systematic misinterpretation. Predictive ML seeks to maximize generalization performance on unseen data. Typical examples include diagnostic support models^31)^ and prognostic prediction models.^32)^ Their performance is primarily evaluated using metrics such as the area under the curve and accuracy on external validation datasets. This represents the dominant paradigm in current medical ML research. In contrast, exploratory ML refers to the use of ML methods with the primary objective of identifying latent structure and generating hypotheses, rather than optimizing predictive performance.^33)^ Its goal is to maximize stability and interpretability, with the focus on revealing latent structures and generating hypotheses from them rather than on predictive accuracy. Thus, the evaluation criteria must differ. Rather than predictive performance, it is important whether the identified structures are reproducible across different data splits or analytical methods and whether the interpretations are consistent with existing biological knowledge.

**Table 1:** Definition of two types of machine learning

Predictive ML	Machine learning is designed to maximize predictive performance on unseen data, which is evaluated by generalization metrics such as accuracy or AUC.
Exploratory ML	Machine learning is designed to identify underlying structures and relationships in data to generate hypotheses, rather than to optimize predictive performance.

AUC: area under the curve

Explainable AI focuses on interpreting predictions generated by predictive models, whereas exploratory ML aims to derive new structural hypotheses independent of predictive tasks (**Table 2**). These 2 roles are not mutually exclusive. A single analysis may serve both predictive and exploratory purposes.^34,35)^ In such cases, it is essential to distinguish the evaluation criteria according to the intended objective. The methods themselves are not the issue. The problem is that analyses are often designed and reported without clearly defined objectives. Biomarker discovery can be viewed as a typical application of exploratory analysis; however, it is often framed as outcome-driven feature selection rather than structural pattern discovery (**Table 2**).

**Table 2:** Conceptual distinctions between exploratory machine learning and related approaches

Concept	Definition	Similarity to exploratory ML	Difference from exploratory ML	Examples
Exploratory Data analysis	Exploratory data analysis focuses on summarizing and visualizing data to identify patterns and generate initial insights before formal confirmatory modeling.	Both aim to explore data and generate hypotheses when predefined hypotheses or model structures are incomplete or unavailable.	EDA primarily relies on descriptive and visualization techniques, whereas exploratory ML enables the identification of complex structural relationships using algorithmic methods.	Data visualization, summary statistics, scatter plots, principal component analysis
Explainable AI	Explainable AI focuses on interpreting and explaining the outputs of predictive models to improve transparency and trust.	Both emphasize interpretability and aim to extract meaningful insights from data.	Explainable AI depends on predictive models, whereas exploratory ML focuses on discovering structures independent of predictive tasks.	SHAP, LIME, saliency maps, feature importance analysis
Biomarker discovery	Biomarker discovery aims to identify variables associated with specific clinical outcomes using statistical or machine learning methods.	Both involve identifying relevant features and patterns within complex datasets.	Biomarker discovery is typically outcome-driven, whereas exploratory ML can identify structures and relationships without predefined outcomes.	Gene expression signature discovery, protein biomarker identification, radiomic feature selection, metabolomic marker screening
Hypothesis-generating study	A hypothesis-generating study is a research design aimed at proposing new hypotheses based on observed data rather than testing predefined ones.	Both are oriented toward generating hypotheses from data.	Hypothesis-generating studies define the purpose of research, whereas exploratory ML provides a methodological framework for conducting such analyses.	Case reports/case series, exploratory subgroup analysis, descriptive epidemiology, EDA

ML: machine learning; EDA: exploratory data analysis; AI: artificial intelligence; SHAP: Shapley additive explanations; LIME: local interpretable model-agnostic explanations

Methods such as clustering, dimensionality reduction, and network analysis have long been used in biomedical research. However, they have rarely been explicitly positioned within the framework of exploratory ML. As a result, analyses aimed at hypothesis generation have often been misinterpreted as failures of predictive modeling.

## Why Exploratory ML is Needed in Medicine

In medicine, conventional statistical approaches are often insufficient to address structural complexity in clinical data, creating a clear need for exploratory ML. First, there are organs in which direct in vivo validation is not feasible. In regions such as the aorta or the brain, biopsy has been difficult due to ethical and technical constraints.^36)^ In such settings, disease mechanisms must be inferred from indirect data, including noninvasive imaging, angioscopy, and clinical course.^37)^ These data are multilayered and often exhibit nonlinear relationships. As a result, hypothesis-driven approaches struggle to capture the overall structure. Exploratory ML enables the identification of latent structures from observational data and enables hypothesis generation even in areas where direct validation is difficult. Second, in emerging diseases or with novel diagnostic modalities, the clinical significance of findings is often not well defined. For example, spontaneously ruptured aortic plaques and related injuries represent a set of observations whose interrelationships remain unclear.^36,37)^ In such cases, there is no clear starting point for hypothesis-driven analysis. Exploratory ML can visualize co-occurrence patterns and relationships within the data, thereby supporting hypothesis generation at an early stage of investigation. These limitations collectively demonstrate that exploratory ML is not optional but necessary in certain areas of medical research.

## Methodological Approaches

Representative ML techniques for predictive and exploratory purposes are summarized in **Table 3**.

**Table 3:** Representative machine learning techniques

Predictive ML
Logistic regression
Random forest
XGBoost/gradient-boosted trees
Support vector machine
Neural networks/deep learning
Decision tree
k-nearest neighbors
Naive Bayes
Cox model/penalized Cox
LightGBM/CatBoost
Exploratory ML
Hierarchical clustering
k-means clustering
DBSCAN/density-based clustering
PCA
t-SNE
UMAP
Network analysis
LASSO/Elastic Net (feature selection)
SHAP (feature importance)
Consensus clustering

ML: machine learning; XGBoost: Extreme Gradient Boosting; LightGBM: Light Gradient Boosting Machine; CatBoost: Categorical Boosting; DBSCAN: density-based spatial clustering of applications with noise; PCA: principal component analysis; t-SNE: t-distributed stochastic neighbor embedding; UMAP: uniform manifold approximation and projection; LASSO: least absolute shrinkage and selection operator; SHAP: Shapley additive explanations

### Unsupervised learning

Unsupervised learning is a central component of exploratory ML, as it enables structure discovery without predefined labels.^38)^

Common clustering methods include k-means,^39)^ hierarchical clustering,^40)^ and density-based spatial clustering of applications with noise (DBSCAN).^41)^ Principal component analysis (PCA),^42)^ t-distributed stochastic neighbor embedding (t-SNE),^43)^ and uniform manifold approximation and projection (UMAP)^44)^ are commonly used for dimensionality reduction. These methods project high-dimensional data into a lower-dimensional space. This allows visualization of global structure, outliers, and potential subgroups. In particular, t-SNE and UMAP are effective in preserving nonlinear relationships. They are useful for exploring structure in clinical and omics data.

However, unsupervised learning also has important limitations. Clustering results depend on the choice of algorithm and hyperparameters. Therefore, stability should be evaluated, for example, by bootstrap methods.^45)^ In addition, low-dimensional visualization reflects only 1 aspect of the data. It should not be over-interpreted as a direct representation of reality. These results should be regarded as a starting point for hypothesis generation.

### Supervised learning with interpretation

Supervised learning is originally designed to maximize predictive performance. It can also function as an exploratory ML approach when combined with interpretability methods. Here, prediction is not the goal; rather, the aim is to extract relationships among variables that the model has learned.

Shapley additive explanations (SHAP) and permutation importance are commonly used to quantify the contribution of each feature to model output. SHAP is based on Shapley values from game theory and provides an additive decomposition of feature contributions.^46)^ Permutation importance evaluates the decrease in model performance when a feature is randomly shuffled.^47)^ This provides an intuitive measure of feature importance. These methods can identify relevant features that may not be detected by conventional univariate analysis.

Regularization methods such as the least absolute shrinkage and selection operator (LASSO)^48)^ and Elastic Net^49)^ shrink the coefficients of less relevant variables toward zero. This leads to sparse models. The process of selecting important variables from many candidates can be viewed as a form of hypothesis generation.

These approaches, however, also have inherent limitations. SHAP values reflect contributions within a model and do not imply causal relationships. In addition, feature importance rankings may differ across models, such as Random Forest^47)^ and Extreme Gradient Boosting (XGBoost)^50)^, even when applied to the same dataset. Therefore, stability is not guaranteed. Sensitivity analyses using multiple models, as well as bootstrap-based confidence intervals for feature importance, are recommended.

### Network analysis

Network analysis provides a powerful framework for representing complex relationships among variables as structured systems.^51,52)^ The method plays a central role in exploratory ML. Each feature is represented as a node, and correlations or co-occurrences as edges. Network analysis allows visualization of interactions within the data and facilitates the identification of structural patterns that cannot be detected by examining variables individually. In medicine, network analysis has been applied to disease networks,^53)^ gene expression networks,^54)^ and symptom co-occurrence networks.^55)^ Network metrics such as degree centrality, betweenness centrality, and clustering coefficient can be calculated. These measures are useful for identifying important hubs and functional clusters in disease. In addition, module analysis can detect subnetworks in which multiple variables change together. Network analysis may contribute to a better understanding of multifactorial disease mechanisms.

In conventional multivariable analysis, the choice of variables often depends on prior knowledge or subjective judgment. As a result, higher-order interactions among variables may be overlooked. Network analysis provides a data-driven approach to address this limitation. Providing a data-driven overview of relational structure can offer a rational basis for variable selection before formal multivariable modeling.

Network structure depends on how edges are defined, such as the threshold used for correlation coefficients. In addition, correlation-based networks are affected by confounding. Therefore, observed edges do not necessarily represent true biological relationships. These results should be interpreted as tools for hypothesis generation rather than confirmatory evidence.

### Data handling

Data preprocessing and sampling methods can fundamentally influence exploratory results. This is especially important when class imbalance is present. In such cases, the features of the minority class may not be sufficiently reflected in the data structure, and the extracted patterns may become biased.^56)^

Oversampling methods such as synthetic minority over-sampling technique (SMOTE) are designed to reduce class imbalance by generating synthetic samples through linear interpolation in the feature space of the minority class.^57)^ In predictive ML, this may improve generalization performance. By contrast, different considerations are needed in exploratory ML. Because synthetic samples may alter the original distributional structure of the data, the structures identified by clustering or network analysis may be artificially distorted. As a result, the generated hypotheses may not fully reflect the original data structure.

Therefore, when SMOTE is used in exploratory ML, its effect should be evaluated explicitly. For example, it is useful to compare structural stability before and after oversampling, or to present results from the original data alone as a sensitivity analysis. The use of SMOTE may be acceptable as an initial exploratory step, but its impact on the data structure must be carefully evaluated.

### Differences from conventional statistics

Many conventional statistical analyses in clinical research are designed to test predefined hypotheses and draw conclusions through p-values and confidence intervals, whereas ML extracts patterns directly from data. Whereas conventional analysis begins with a hypothesis, it generates one as an outcome of the process. This difference reflects not only a methodological distinction but also a fundamental difference in how knowledge is generated.

In conventional statistics, the validity of a study is assessed by controlling type I error (α), applying multiple comparison corrections (such as Bonferroni or Benjamini–Hochberg), and ensuring sufficient statistical power. These approaches are intended to reduce the risk of falsely supporting a predefined hypothesis. By contrast, such statistical criteria are not directly applicable to exploratory ML. Stability is evaluated using cross-validation, and uncertainty is assessed using bootstrap-based confidence intervals.

The 2 approaches also differ in interpretability. In linear regression or generalized linear models, coefficients are directly interpretable as effect sizes under fixed conditions of other variables. Nevertheless, feature importance in models such as Random Forest or XGBoost represents the contribution of variables to the overall prediction. It does not provide a direct interpretation as an effect size.

This asymmetry in evaluation and interpretation is a fundamental source of mismatch during peer review. Reviewers trained in conventional statistics often criticize ML-based exploratory studies for lacking p-values or multiple comparison correction. On the other hand, ML researchers may criticize such studies for lacking methodological novelty.^58)^ Explicitly separating these perspectives and adopting evaluation criteria appropriate to the research objective is an important first step toward resolving this issue.

In exploratory ML, evaluation should focus on the robustness and reproducibility of identified structures rather than on predictive accuracy alone. Several practical approaches may be useful for this purpose. Cluster stability can be assessed using resampling or bootstrap-based methods. The robustness of feature importance or network structures can be evaluated across different preprocessing strategies, model choices, or analytical pipelines. Sensitivity analyses may help determine whether identified patterns remain consistent under alternative parameter settings or data transformations. In addition, replication of identified structures in external datasets or independent cohorts may strengthen the credibility of exploratory findings. Although no single standard currently exists, these approaches may provide a practical framework for evaluating exploratory analyses.

### Relationship with existing guidelines

Current reporting guidelines, such as TRIPOD-AI^59)^ and STARD-AI,^60)^ are widely used. TRIPOD-AI aims to ensure transparent reporting of prediction model development and validation. It focuses on assessing generalization performance and external validation. STARD-AI extends the original STARD guideline for diagnostic accuracy studies to AI-based tools. It emphasizes standardized reporting when comparing AI systems with reference standards.

Still, these guidelines are designed primarily for confirmatory and performance-oriented research. They do not explicitly address exploratory analyses aimed at hypothesis generation. To properly evaluate exploratory ML, new reporting frameworks are needed. These should focus not on predictive accuracy, but on structural stability, reproducibility, and interpretability.

### Position as a hypothesis-generating study

Exploratory ML can be positioned as a hypothesis-generating study. Results should be presented as structural hypotheses that guide subsequent validation, rather than as definitive conclusions. ML should be understood not as providing final evidence but as a method for systematically generating testable questions. Hypothesis-generating studies are placed upstream of confirmatory studies in the hierarchy of clinical evidence. Randomized controlled trials and prospective cohort studies represent confirmatory inference, whereas exploratory studies define what should be tested. Exploratory ML belongs to this upstream stage. Its value lies not in predictive performance but in the structural validity and testability of the generated hypotheses.

Hypotheses derived from ML should be explicitly formulated. For example, results should be expressed as “this pattern suggests the hypothesis that …”. The required data, study design, and validation criteria should also be clearly described. Incorporating the processes of exploration, hypothesis formulation, and validation design into the manuscript allows exploratory ML to be recognized as an independent contribution rather than a preliminary analysis.

Positioning exploratory ML as a hypothesis-generating approach reduces the risk of overinterpretation. By clearly stating that the findings are hypotheses, the risk of misinterpreting associations as causal explanations can be minimized. This transparency provides a shared framework for readers, reviewers, and clinicians, and improves the credibility of ML-based research.

### Interpretation and Pitfalls

The ongoing debate over whether ML outperforms conventional methods is fundamentally misdirected in the context of exploratory analysis.^61)^ In contrast, this debate is limited to comparisons of predictive performance. In the context of exploratory ML, the question of superiority may not be appropriate. The relevant question is which method provides structural insight for a given clinical problem. Interpretability requires the same caution. SHAP values and permutation importance indicate which variables a model prioritized during prediction. They reflect model-specific associations rather than causal relationships. Although this distinction appears obvious, it is frequently overlooked in practice. Important features are often read as causal factors of disease. Authors may also describe them in that context without awareness. The same applies to structures visualized in network analysis. Edges represent correlations or co-occurrence relationships. They do not directly reflect functional connections in biological systems. When relationships influenced by confounding or measurement bias appear as structures in a network, they may be artifacts of the data. This issue becomes particularly pronounced in small datasets. Explainability and interpretability are not synonymous. A model that is explainable is not necessarily interpretable. Visual explanations provided by explainable artificial intelligence are intuitively persuasive, but that persuasiveness does not guarantee a correct understanding of the underlying mechanism. This “explainability trap” represents a critical risk of overconfidence in clinical applications. To avoid these pitfalls, exploratory ML results should be evaluated based on the stability of patterns across multiple methods and data splits, consistency with established biological knowledge, and the ability to generate testable hypotheses. Only findings that meet these criteria can be considered reliable inputs for subsequent confirmatory research.

## Future Directions

A central challenge for future research is establishing a clear transition from exploratory analysis to confirmatory research, including the validation of structural hypotheses in independent datasets or prospective studies. Exploratory ML also has particular value for community hospitals and smaller institutions. Structural pattern extraction and hypothesis generation are feasible even with limited sample sizes and imbalanced data. This presents a different model of research from the conventional framework centered on large-scale studies. This may expand opportunities for generating new knowledge from pre-hypothesis clinical observations. Establishing a standardized framework for evaluating exploratory ML will be essential for its acceptance in clinical research. Notably, ML is quite difficult for many medical researchers, partly due to the limited availability of practical guidance and the technical barriers associated with tools such as R (R Foundation for Statistical Computing, Vienna, Austria) and Python (Python Software Foundation, Beaverton, OR, USA). To enable broader adoption, simpler and more accessible analytical approaches are required.

However, these challenges are not merely technical but also conceptual. Prediction remains an important objective in many clinical applications. The issue is not prediction itself, but the implicit assumption that predictive performance constitutes the primary criterion for evaluating ML-based studies. This perspective risks underestimating the value of exploratory analyses.

As pointed out in previous studies,^62,63)^ clinical medicine has been predominantly focused on outcomes.

In this context, it is equally important to reveal underlying structures and generate clinically meaningful hypotheses.

## Conclusion

ML in medicine has been predominantly framed as a predictive tool. This review defines a distinction between predictive ML and exploratory ML. Exploratory ML prioritizes the identification of structure and hypothesis generation rather than predictive performance. I argue that evaluation criteria must be aligned with the primary objective of the analysis. Exploratory ML should be assessed based on the stability, reproducibility, and interpretability of the identified structures. Recognizing this distinction provides a foundation for improving study design, interpretation, and peer review in medical research. Without this conceptual distinction, exploratory studies may be misinterpreted as poorly performing predictive models. As a result, clinically meaningful insights may be systematically overlooked.
